# Mr. Richmond on Cataract in India

**Published:** 1826-07

**Authors:** 


### MR. RICHMOND ON CATARACT IN INDIA.

In the May Number of the Medical and Physical Journal, is a paper by Mr. Richmond, of the 4th Light Dragoons, on Cataract, &c. in India, which shews in so favourable a light both the *surgeon* and the *man*, that we shall give it all the circulation our pages can afford.

The report is dated at Poonah, from the 6th May to the 12th December, 1824. In that space of time 504 were restored to sight; of these 407 were cataracts, 9 artificial pupils and 2 pterygiums. A very great number of these can read well without the aid of glasses, many would be benefited by them, as they have the flattened cornea of old age; some, 29, lost their sight afterwards by their imprudence and intolerance of restraint, which is a very great obstacle to success in operations on the natives of India. Our author remarks, that to manage these people with ease, light must be given in one instant, and with as little pain as possible, he further observes, that couching may be performed without the patient suffering more than he does in blood-letting, scarcely so much. It was our author's custom to go round through town and village seeking the blind, that he might restore them their sight. One evening he went down to the river-side, to operate on an old blind woman. She had a cataract in her right eye, this was removed in an instant, and her sight given back to her. On his return he was surrounded by halt and lame and blind; and before dark, nine cataracts were removed. His fame spread, and patients came from all the country round, to the distance of 150 miles to visit him. Our author too, journeyed to considerable distances to effect his benevolent mission. He rode a course of fifty miles to Telligahum and the adjacent parts, where he operated with success on 27 cataracts; and on 222 with the same result at Admednuggur, in the short space of two months; four artificial pupils too were made here. The history of a boy, twelve years of age, who had a new pupil cut out after eight years blindness, reminds us strongly of Honoré Trezel, who our readers will, perhaps, recollect had his hearing restored by an operation. This child, on receiving vision, was so overjoyed at the delightful change from utter darkness to the pleasant light of day, that he tore about the place half mad, took up every thing he could reach, examining it with the hand and eye, with insatiable curiosity. Another boy, who was almost congenitally blind, on having vision restored, was at first quite stupid, and it was some time before he became accustomed to this new sensation. To pursue the thread of our narrative. Our author would journey thirty and forty miles a day, to restore sight to the inhabitants of distant places, and return, loaded with blessings, some literally "laying hold of the hem of his garments," with every expression of gratitude. Almost all classes of people were equally liable to cataract; if any thing the peasants, artisans, and coolies more so than the higher ranks. The disease was equally prevalent in country and town. To some, after operation, our author found the common spectacles, ground so as to throw the focus considerably back, of very great service. On the 7th December, 1824, our author received a letter from the Honourable the Governors in Council through the Medical Board, requiring him to instruct the native practitioners in the various operations on the eye. This he did, and eight Mussulman Doctors attended his lectures with considerable diligence, particularly on operation days, which proves, we think, that the love of the knife is not confined to England. The native mode of operating for cataract is clumsy enough. It is this: with their instrument, an old rusty lancet, the point broken off, and coarsely ground to an oval form, they pierce the lower hemisphere of the globule behind the iris, then pass a triangled copper probe through the lacerated orifice into the eye, and endeavour to depress the cataract. In old people they succeed once in seven or eight times, but where the lens and its capsule have a diseased attachment, and the vitreous cells are unbroken, their prospect of success is feeble indeed. If they do not bring on inflammation, they frequently induce amaurosis. This statement is in contradiction to the idea which has got abroad, that these rude operators on the eye are very fortunate.

In conclusion, we must beg to tender our meed of applause, humble though it be, to the author of this paper. Had a man appeared four centuries ago, whether on the banks of the Ganges or the Tiber, and worked what he has worked, assuredly he would have been dignified by the vulgar, as a prophet or a saint. As it is, though we fear he cannot hope for canonization, yet he has, what is, perhaps, as valuable, the applause and approbation of his brethren. Indeed, seeing what we see, and hearing what we hear every day, it is pleasant to turn from members who disgrace the profession, to which alas! they belong, to those who, like Mr. Richmond, give it a character in the world's eye for benevolence and good-will.

---

